# Supplementary material for: Gut-Faecal Microbial and Health-Marker Response to Dietary Fumonisins in Weaned Pigs
Source: Toxins (Basel). 2023 May 11;15(5):328. doi: 10.3390/toxins15050328 (PMC10222793; doi:10.3390/toxins15050328)
Supplement: Supplementary file 1 [file toxins-15-00328-s001.zip › toxins-2254954-supplementary.pdf]

SUPPLEMENTARY MATERIAL

Supplementary Table S1. Effects of dietary FBs on the antioxidant parameters glutathione (GSH) and glutathione peroxidase (GSHPx), and the end-product of lipid peroxidation, malondialdehyde (MDA) in weaned pigs (data are means ± standard deviation (SD) of 6 individuals/group)

**Parameter**	**Control (G1)**	**15 mg/kg FBs (G2)**	**30 mg/kg FBs (G3)**	***p*- Values**
Kidney				
GSH, µmol/g	14.1 ± 0.7	15.2 ± 0.7	15.9 ± 2.3	0.13
GSHPx, IU/g prot.	2.6 ± 0.9	2.7 ± 0.6	3.2 ± 1.7	0.63
MDA, nmol/g	29.2 ± 11.6	34.2 ± 7.3	31.6 ± 5.1	0.60
Liver				
GSH, µmol/g	7.7 ± 0.6	7.5 ± 1.1	7.7 ± 1.2	0.93
GSHPx, IU/g prot.	4.1 ± 0.4	3.9 ± 0.6	4.0 ± 0.5	0.66
MDA, nmol/g	71.0 ± 7.8	65.9 ± 12.0	59.6 ± 9.4	0.26
Lung				
GSH, µmol/g	4.9 ±0.9	4.78 ± 0.5	4.9 ± 0.9	0.91
GSHPx, IU/g prot.	5.4 ± 0.5	5.5 ± 0.5	5.8 ± 0.5	0.28
MDA, nmol/g	41.6 ± 7.7	43.0 ± 7.1	36.6 ± 2.7	0.20
Plasma				
GSH, µmol/g	2.6 ± 0.2	2.6 ± 0.3	2.4 ± 0.3	0.31
GSHPx, IU/g prot.	4.6 ± 0.5	4.3 ± 0.3	4.0 ± 0.8	0.09
MDA, nmol/g	4.9 ± 0.8	6.1 ± 1.4	6.1 ± 1.4	0.16

SUPPLEMENTARY MATERIALS

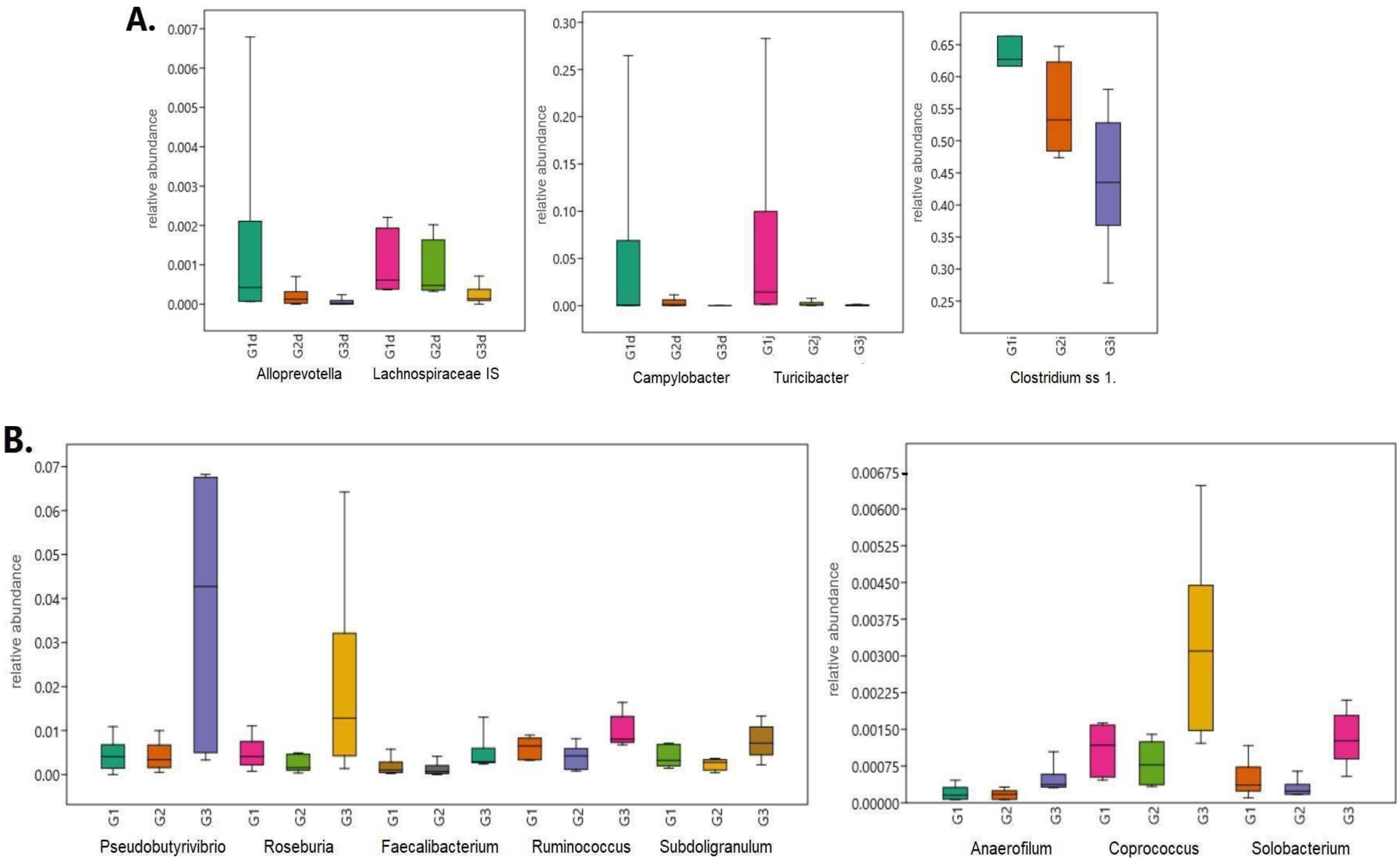

Supplementary Figure S1. Changes in the mean relative abundances of genera listed in Table 3. between the three treatments (G1, G2 and G3), where boxplots show relative abundances for the six animals analyzed in a group. **(A)** Significant changes at the genus level in the small intestine: duodenum (d), jejunum (j), ileum (i) in the three treatment groups (G1, G2 and G3). **(B)** Significant changes at the genus level in the day 21 faecal samples. Please see Table 3. and the text for further details.

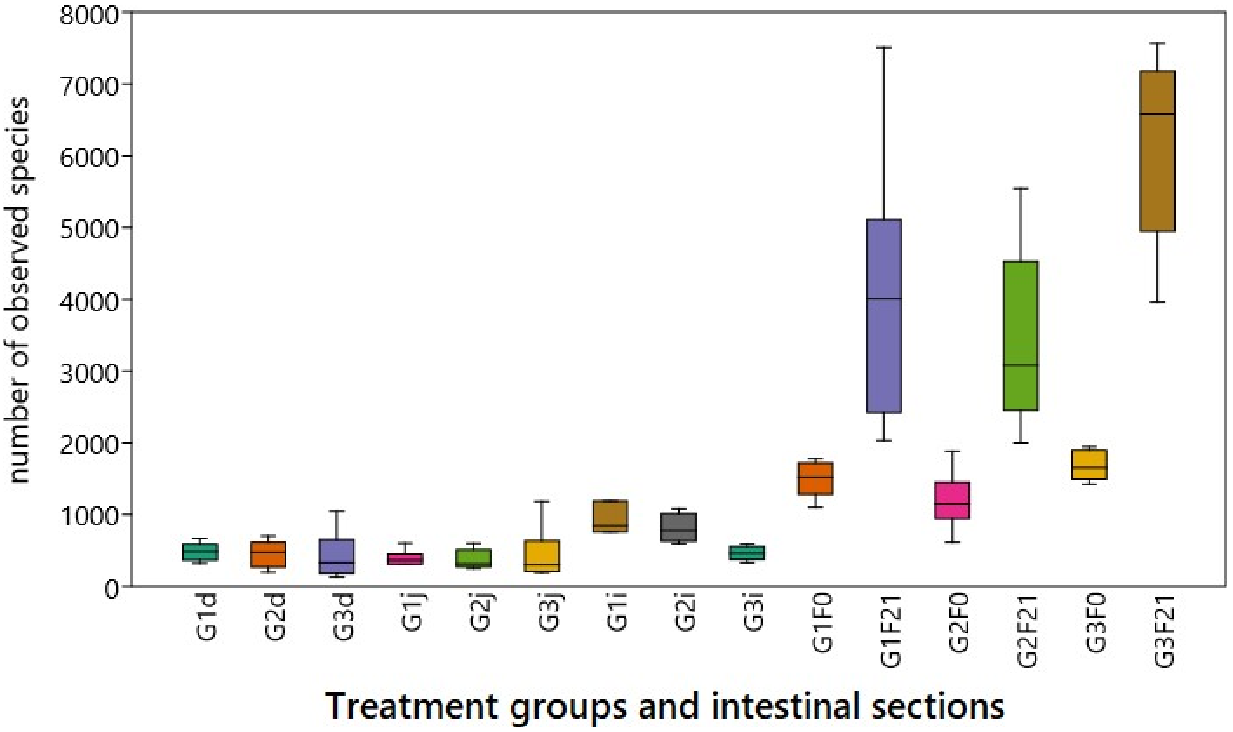

Supplementary Figure S2. The number of observed species for pig intestinal samples in three treatments (G1, G2 and G3), where boxplots show the number of observed species for the six animals analyzed in a group as calculated by the QIIME pipeline. Duodenum (d), jejunum (j), ileum (i), day 0 faeces (F0) and day 21 faeces (F21) in the three treatment groups (G1, G2 and G3).

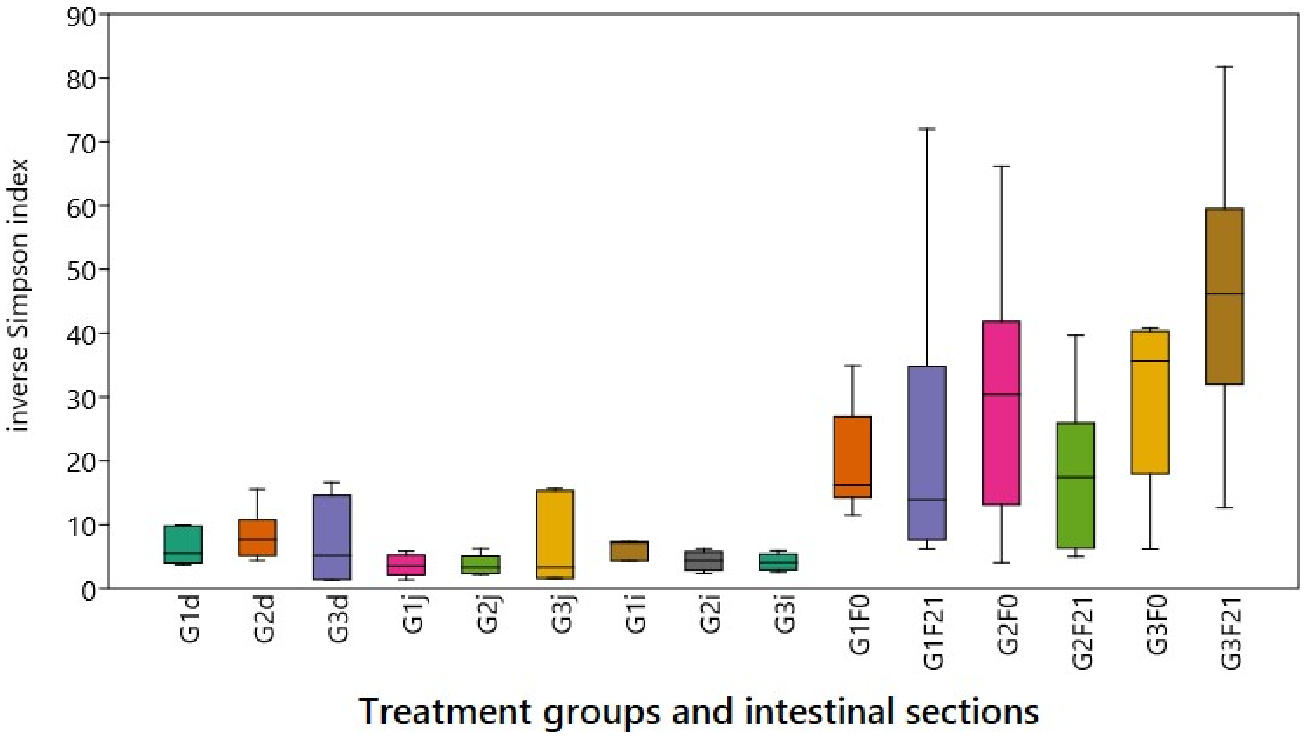

Supplementary Figure S3. The inverse Simpson index for pig intestinal samples in three treatments (G1, G2 and G3), where boxplots show the inverse Simpson indices for the six animals analyzed in a group as calculated by the QIIME pipeline. Duodenum (d), jejunum (j), ileum (i), day 0 faeces (F0) and day 21 faeces (F21) in the three treatment groups (G1, G2 and G3).

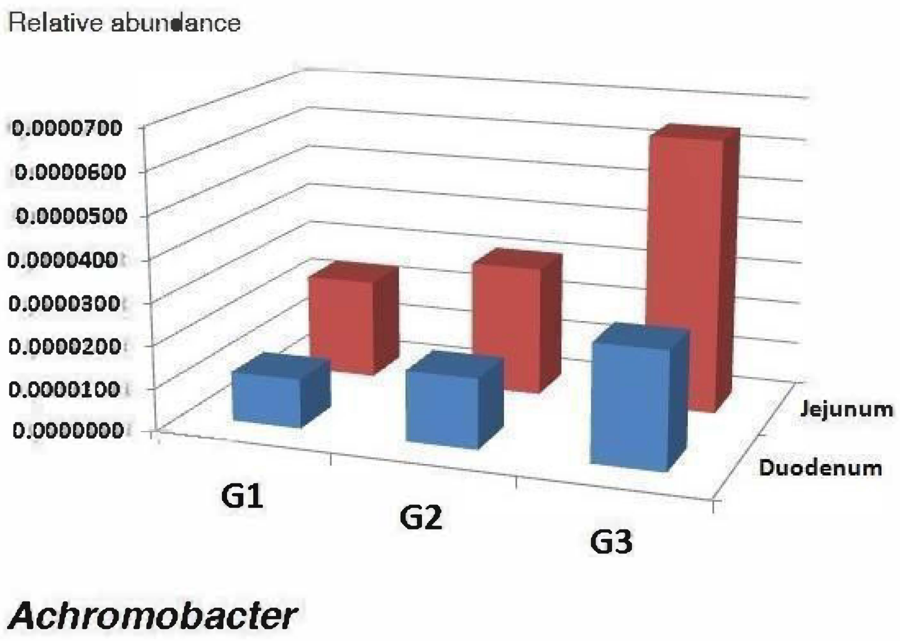

Supplementary Figure S4. Mean relative abundances of the genus *Achromobacter* in the three treatment groups (G1, G2 and G3) among the six animals analyzed in a group in the intestinal section's duodenum and jejunum.

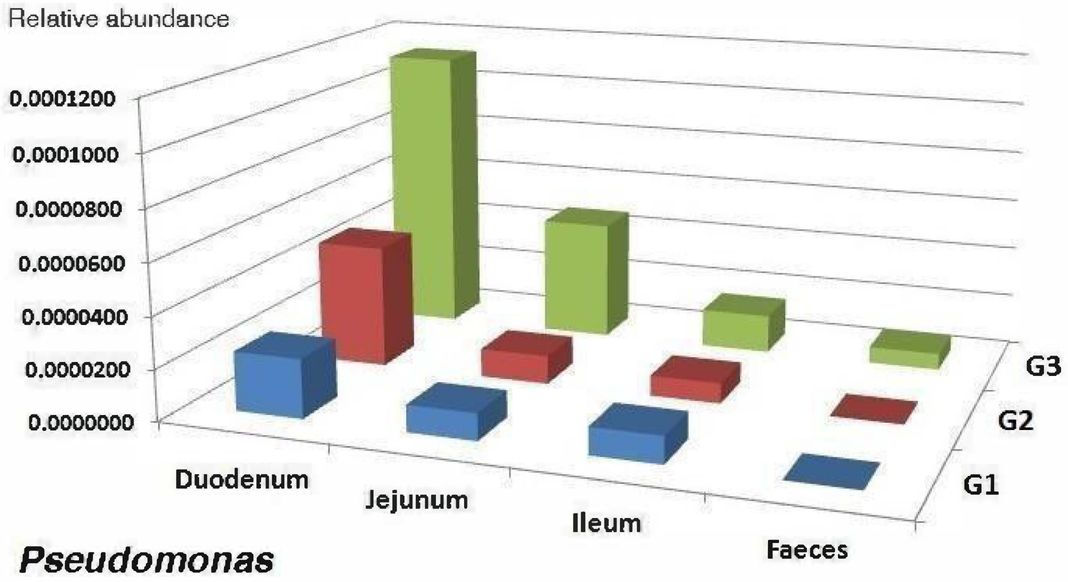

Supplementary Figure S5. Mean relative abundances of the genus *Pseudomonas* in the three treatment groups (G1, G2 and G3) among the six animals analyzed in a group in the examined intestinal sections.

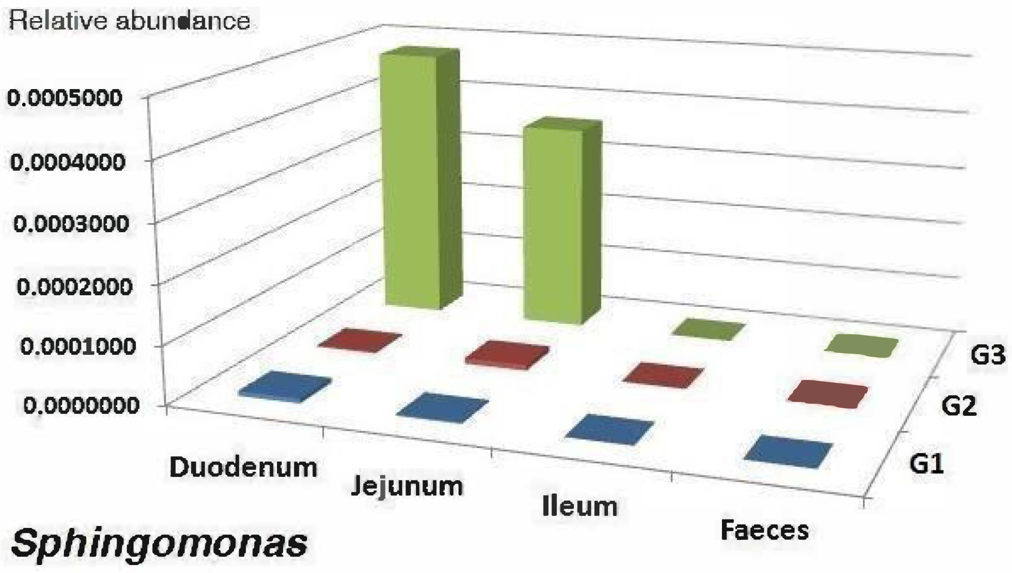

Supplementary Figure S6. Mean relative abundances of the genus *Sphingomonas* in the three treatment groups (G1, G2 and G3) among the six animals analyzed in a group in the examined intestinal sections.

SUPPLEMENTARY MATERIALS

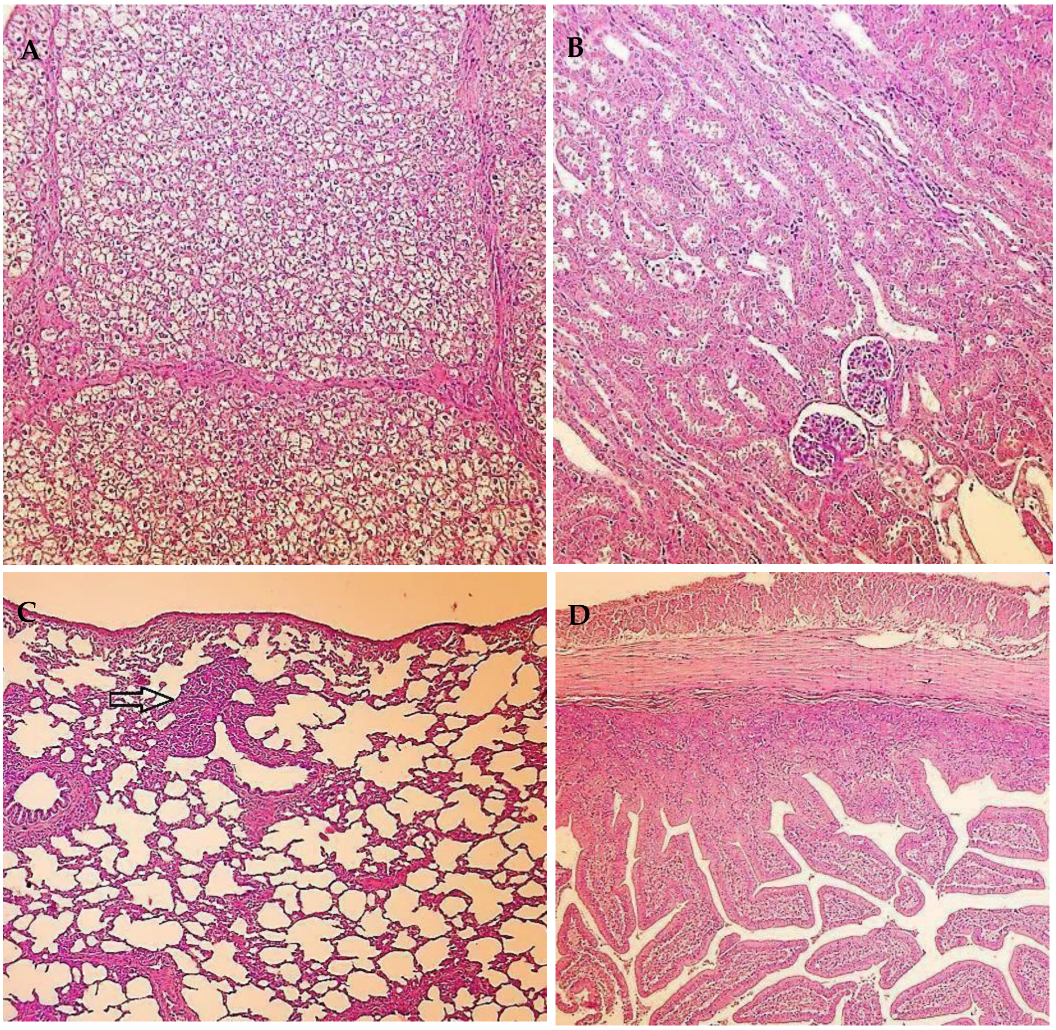

Supplementary Figure S7: Histological illustrations of organs of a healthy pig **(Control; G1)** for 21 days. **(A)** Slight vacuolation of hepatocytes in the liver (H.-E., 200x), **(B)** Some exfoliated cells in the tubular lumen of the kidney (H.-E., 200x), **(C)** Focal mononuclear cell infiltration in the interalveolar saepta of the lung (arrow) (H.-E., 100x), **(D)** Slight mononuclear cell infiltration in the villi of the jejunum (H.-E., 100x)

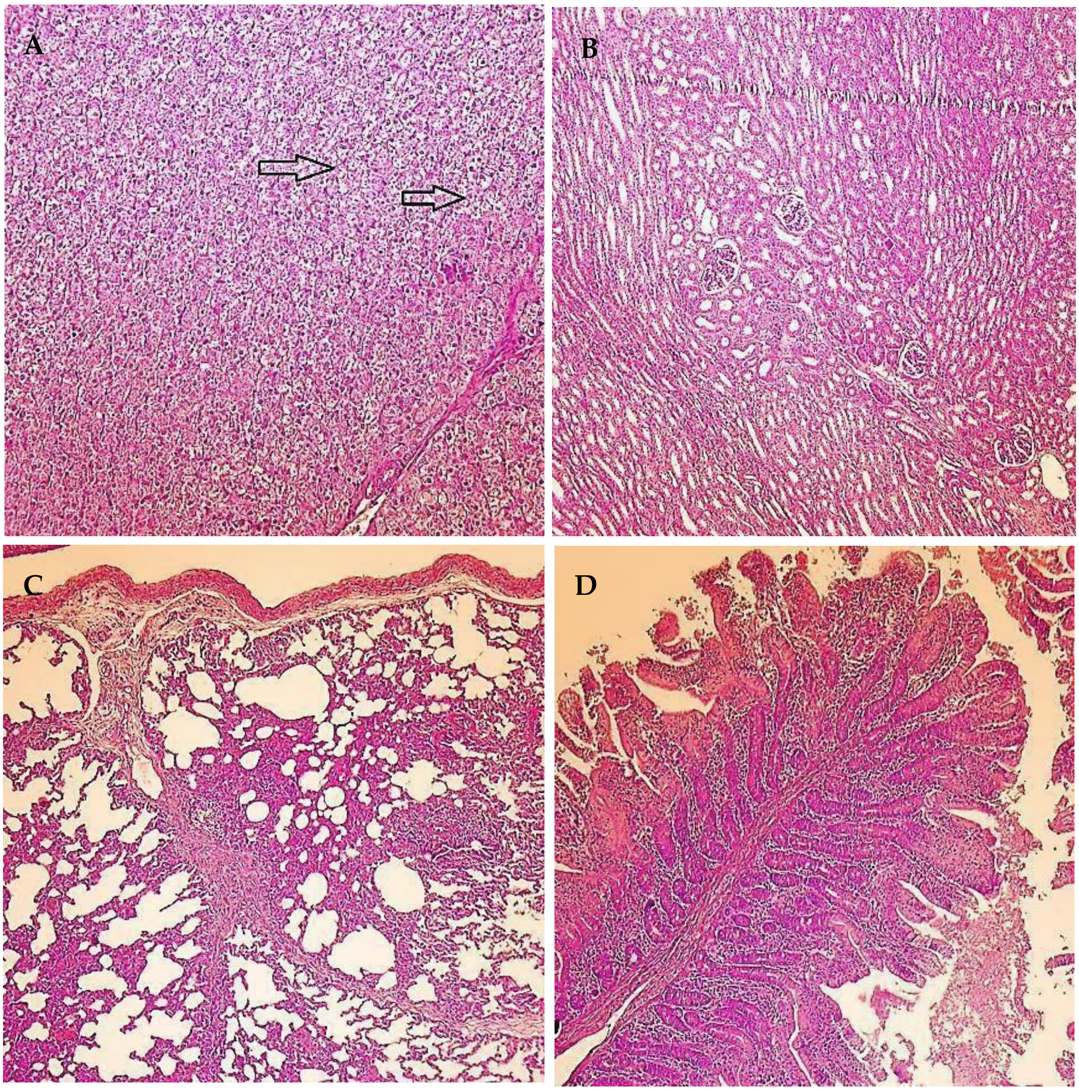

Supplementary Figure S8: Histological illustrations of organs of a pig that consumed **15 mg/kg FBs (G2)** diet for 21 days. **(A)** Vacuolation of hepatocytes, multifocal lysis and necrosis of hepatocytes (arrow), (H.-E., 200x) of the liver, **(B)** Some exfoliated cells in the tubular lumen of the kidney (H-E., 200x), **(C)** Multifocal mononuclear cell infiltration in the interalveolar saepta of the lung (H.-E., 100x), **(D)** Slight mononuclear cell infiltration in the villi (H.-E., 100x) of the small intestine

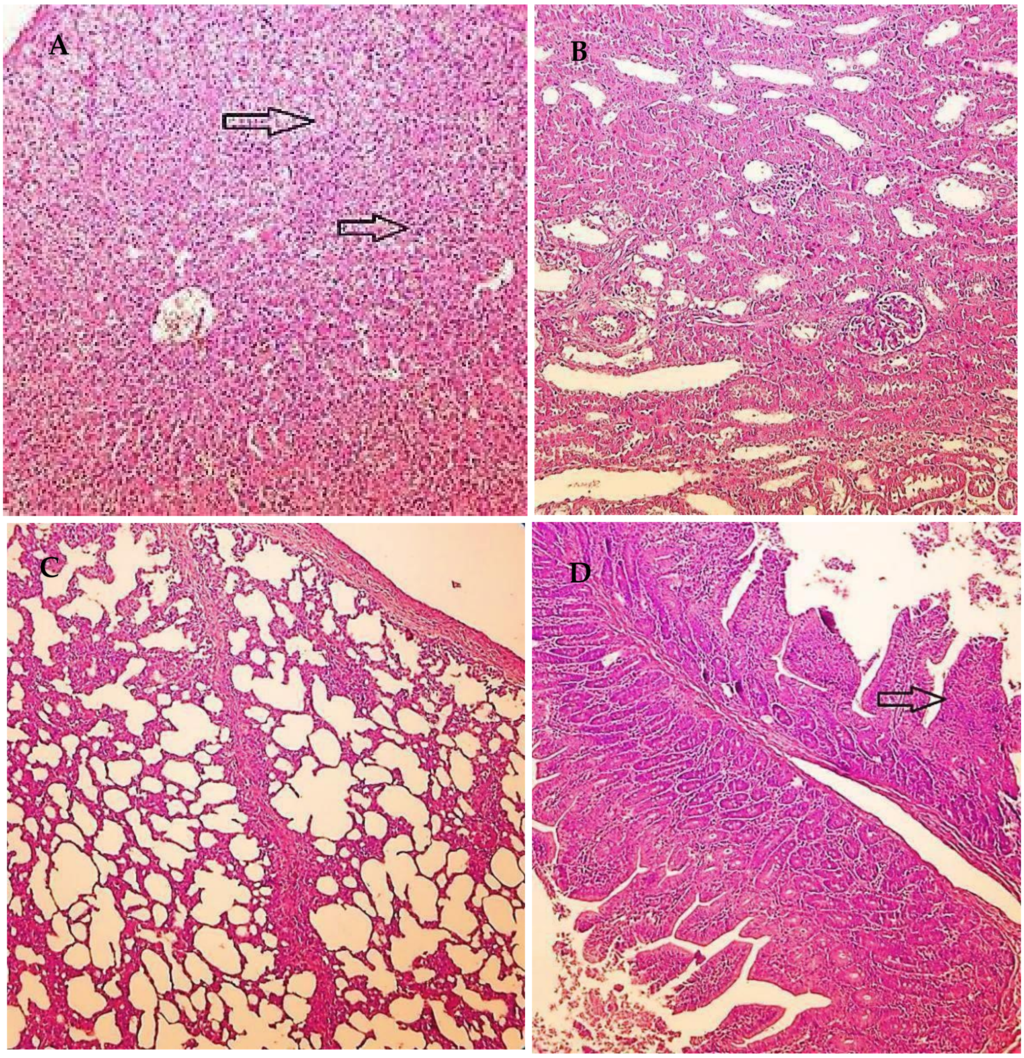

Supplementary Figure S9: Histological illustrations of organs of a pig that consumed **30 mg/kg FBs (G3)** diet for 21 days. **(A)** Multifocal lysis, necrosis of hepatocytes (arrow), and multifocal swelling and proliferation of mononuclear phagocyte system (MPS)- cells (arrow) of the liver (H.-E., 200x), **(B)** Some exfoliated cells in the tubular lumen, and dilatation of some tubuli in the kidney (H.-E., 200x), **(C)** Mononuclear cell infiltration in the interalveolar saepta of the lung (H.-E., 100x), **(D)** Mononuclear cell infiltration and thickening (arrow) in the villi of the small intestine (H.-E, 100x)
